# The role of informal carers in the diagnostic process of heart failure: a secondary qualitative analysis

**DOI:** 10.1186/s12872-019-1075-6

**Published:** 2019-04-23

**Authors:** Frances Bell-Davies, Clare Goyder, Nicola Gale, F. D. Richard Hobbs, Clare J. Taylor

**Affiliations:** 1 Nuffield Department of Primary Care Health Sciences, University of Oxford, Radcliffe Primary Care building, Radcliffe Observatory Quarter, Woodstock Road, Oxford, OX2 6GG UK; 2 Health Services Management Centre, University of Birmingham, Birmingham, UK

**Keywords:** Heart failure, Diagnosis, Carer, Symptoms, Awareness

## Abstract

**Background:**

Heart failure (HF) is a common clinical syndrome, particularly in older people, and symptoms can develop gradually. The aim of this study was to explore the role of informal carers in the HF diagnostic process.

**Methods:**

Secondary analysis of qualitative interviews with 16 participants with a new diagnosis of HF. Original interviews were conducted in the participant’s home, with carers present in some cases. Interview transcripts were re-analysed using the Framework Method for themes pertaining to informal carers and how they were involved in the diagnostic process.

**Results:**

Informal carers often noticed symptoms, such as breathlessness, before participants. In some cases, carers colluded with participants in normalising symptoms but over time, when symptoms failed to resolve or got worse, they encouraged participants to seek medical help. Adult children of participants commonly initiated help-seeking behaviour. During the diagnostic process, carers coordinated participants’ healthcare through advocacy and organisation. Carers were keen to be informed about the diagnosis, but both participants and carers struggled to understand some aspects of the term ‘heart failure’.

**Conclusions:**

Carers play a crucial role in HF diagnosis, particularly in initiating contact with healthcare services, and should be empowered to encourage people with HF symptoms to seek medical help. Improving public awareness of HF could mean informal carers are more likely to notice symptoms. The important role of carers in supporting the patient’s route to diagnosis should be incorporated into future care pathways and explored in further research.

## Background

Heart failure (HF) is a common clinical syndrome associated with gradual onset of symptoms such as breathlessness, ankle swelling and tiredness, as well as reduced life expectancy [1]. Overall, in developed countries, 1–2% of adults are living with HF and prevalence increases with age [2]. The lifetime risk of HF at 55 years of age is 33% for men and 28% for women [3]. Timely diagnosis is important to facilitate treatments proven to reduce symptom burden, increase patients’ quality of life and improve prognosis [4].

People with HF may have reduced physical capacity [4] and require help from family and friends who take on the role of informal carer [5, 6]. A recent study estimated the costs of informal caregiving for patients with cardiovascular disease to be $61 billion globally in 2015 and projected costs to increase to $128 billion by 2035 [7]. Informal carers are involved in a wide range of caring activities, including ‘visible’ caring, such as managing medication and attending to personal needs, and ‘invisible’ caring, for example monitoring signs and symptoms [8]. This can be a significant physical and psychological burden for carers, and they may require additional support themselves to sustain their vital role [9, 10].

While the importance of carers in supporting people with known HF has been established, there is little data on their role prior to and during the HF diagnostic process. The symptoms of HF are often found in other medical conditions. For example, fatigue can be due to anaemia, an underactive thyroid gland, another medical condition, poor sleep or be a normal occurrence in healthy individuals [11]. Symptoms can also be gradual in onset which can make it more difficult for patients to identify that something is wrong. The assumption that people experiencing symptoms will seek care has previously been challenged [12]. There is often a trigger such as occurrence of an interpersonal crisis, perceived interference with social relations and physical activity, or sanctioning of the fact that ‘something is wrong’ by another person [13]. In other diseases with insidious onset, such as dementia, informal carers play a key role in temporalizing symptomatology and reporting changes in the patient to healthcare professionals which can lead to a diagnosis [14].

The aim of this study was to use an existing qualitative dataset to explore how people with HF and their informal carers perceived the contribution of the carer role to the HF diagnostic process.

## Methods

Secondary analysis was carried out using data from a qualitative interview study called ‘From Breathless to Failure’ [15]. The aim of the original study was to explore the experiences of patients with a recent diagnosis of HF and the findings have been published (Taylor et al., BMJ Open 2017). The study team noted that informal carers were usually present during the interviews and provided crucial input to the construction of the patient story. The full methods of the original study can be found in the published paper. In brief, semi-structured interviews with sixteen patients who had received a diagnosis of HF within the previous year were conducted between October and December 2014. Participants were recruited from a secondary care HF clinic serving a socioeconomically diverse area in central England. The prevalence of HF increases with age, and HF affecting younger people is likely to have a different aetiology, so only people over the age of 55 years was invited to participate in the study. Participants with HF who responded were purposively selected to obtain a maximum-variation sample including a balance of demographic characteristics, such as age, ethnicity and social background. Arrangements were made for the use of interpreters to allow inclusion of non-English-speaking participants. All participants received a patient information sheet and provided written consent. Informal carers were present in ten interviews and their contributions were also recorded. Purposive sampling was undertaken for participants with HF only, and not carers, to align with the aims of the original study to explore patient experience.

Interviews were conducted by CT, a female general practitioner (GP) and clinical researcher trained in qualitative methods. Participants were recruited by letter from a secondary care clinic and were not patients of, or known to, the interviewer. Interviews lasted between 30 and 60 min and followed a topic guide which was developed based on existing literature and reviewed throughout in response to themes that were emerging during the concurrent analysis process. The full topic guide can be found in the online appendix. Interviews were recorded and transcribed verbatim and continued until data saturation around key themes for the original study was reached.

Secondary analysis was undertaken using the Framework Method, a type of thematic analysis, that adopts a pragmatic approach to analysing real world data using a clearly defined technique. It is a systematic approach carried out in 7 stages: transcription, familiarisation with the interview, coding, developing an analytical framework with key categories, applying the analytical framework, charting data into the framework matrix and interpreting the data using analytical memos [16].

The analysis was undertaken by CT and FBD, a female medical student who was not involved in conducting the original analysis. Both researchers independently coded the initial transcripts then met to discuss and develop the working analytical framework; this was then applied by FBD to all the transcripts. Data were summarised and charted into Framework matrices (in NVivo V11 and Microsoft Word). Following this, analytical memos were produced for each category of data, including selecting illustrative quotes. From these categories, four central themes were identified which we judged to best capture the experiences over time of participants and their carers in relation to the carers’ role in the diagnostic process. Our interpretations were informed by the classic social science theory on help-seeking practices (e.g. Zola 1973, Calnan 1983) that stresses the importance of social (non-physiological) triggers for consulting a doctor, such as interference with daily activities and the influence (or ‘sanctioning’) of others in the decision to consult [13, 17].

## Results

Sixteen participants who had recently been diagnosed with HF were interviewed once: fifteen interviews were conducted in the participants’ own homes and one was conducted over the telephone. Of the sixteen participants, ten were accompanied by a relative, nine of whom were spouses and one of whom was the participant’s daughter. All of the accompanying relatives could be considered to be informal carers from the information available in the interviews. When informal carers were not present at the interviews, participants described their involvement in the diagnosis of their condition. Fifteen participants were white British and one participant was of African-Caribbean origin. Participants were from a range of socioeconomic background in the West Midlands. Eleven of the participants were male and five were female; nine of the carers present were female and one was male. The median age of participants was 78.5 years. The average length of the interviews was 42 min. Four key themes were identified in the analysis: Tensions between noticing and normalising (before seeking medical help); From noticing to sanctioning (seeking medical help); Co-ordinating care and self-care (getting the diagnosis) and Making sense of information (understanding the diagnosis). The categories accompanying each theme, along with illustrative quotes, are shown in Tables 1, 2, 3 and 4.

**Table 1:** Categories and original data from the theme ‘Tensions between noticing and normalising (before seeking medical help)’

Category	Data
Noticing symptoms before the participant	*Carer: Walking on the flat you do get breathless, you can’t walk and talk really.*
	*Participant: I never really realised that until these [carers] made me aware of it because it was obviously coming on and it was you, weren’t it? A couple of times you said…*
	*Carer: Well it just as you’re walking round the shops you tell me that we need to slow down or you’re struggling to speak and to walk at the same time. (Participant (P)14)*
Normalising symptoms through comparison with own ageing	*Participant: I was walking a long distance and I needed to stop.*
	*Carer: Of course, as we got older, I’m able to walk really, really well, but I get out of breath when I get to the top of the church road. It’s because it’s a very, very slight incline, but it tells on you. As soon as I get to the shop, I get in the shop, it’s gone. It’s just age, I'm sure. (P6)*
Breakdown of normalisations – sudden onset of severe symptoms	*He wasn’t very well on Sunday morning. He said he couldn’t breathe properly, so I said, “Well, perhaps we’d better get you to the hospital”. I phoned the paramedics. I said he couldn’t breathe, he couldn’t get his breath properly and they said, “We’ll come along”. (Carer of P6)*
Breakdown of normalisations – prolonged symptoms	*The thing is, this cough had gone on for weeks and weeks. I thought, that’s not normal. You can’t go on like that forever, just constant cough. (Carer of P9)*

**Table 2:** Categories and original data from the theme ‘From noticing to sanctioning (seeking medical help)’

Category	Data
Carers role in seeking medical help	*Interviewer: What made you first go to your doctors two and a half years ago?*
	*Participant: [My wife] heard me coughing and took me there. (P9)*
	*… my son he said I’m fed up of this, I’m going to take you to the walk-in centre in [town]. (P16)*
	*My two daughters were here at the time, had come to help the wife and they saw the state I was in and I was in and out of bed and making a mess all over the place. They came and they took me to hospital. (P7)*
Reluctance of participants to follow carer’s advice to seek medical help	*I felt poorly, my sons came on the Saturday, and they said, “We should have an ambulance and go down to the hospital.”…[Patient declined]…I must admit I’m pretty strong willed…on Sunday I felt worse and my granddaughter rang…I said, “I'm going to the doctor’s in the morning” but she rang at seven in the night and she said that I was incoherent…they all suddenly descended here and apparently I was slumped in the chair. (P5)*

**Table 3:** Categories and original data from the theme ‘Coordinating care and self-care (getting the diagnosis)’

Category	Data
Examples of coordinating care	*[To interviewer] The [medical correspondence] is upstairs, so this is from this time last year, really, mainly letters of appointments and everything. But that may give you some idea. (Carer of P9)*
Burden of coordinating care	*Well, of course, if I phoned [GP practice] for [Participant], I always phone a few minutes before 8:30 and you can’t always get anyone and then you phone again and it’s engaged, so you just have to keep trying. (Carer of P6)*
Advocating for participant	*Participant: Yeah, because we did find, you phoned the secretary, didn’t you and you said to her…*
	*Carer: Well I asked what number he was on the list and she said number 16…the following week I thought I’d ring… So I rang her again to see what number he was on the list then and she said number 16 and I said, “Have they not done any operations in the last week?” “Oh yes, loads” she said, “but they were all emergencies”. And I said, “Well I would have considered this as an emergency really, if it’s to do with his heart.” (P12)*
Self-care	*I usually say to him so just sit down and get your breath back. Get yourself settled down, and see how things go, and he usually calms down. (Carer of P13)*

**Table 4:** Categories and original data from the theme ‘Making sense of information (understanding the diagnosis)’

Category	Data
Accompanying participants allowed carers access to information	*Those lectures we went to were very good weren’t they because they gave you some idea of of …what to look for if it started to happen again…It’s going to be there and you’ve got to deal with it in your way haven’t you. So those lectures were very good in the fact that they described everything to you. (Carer of P13)*
Carers’ desire to know more about the diagnosis	*I’m forever nagging you, always asking you what they’ve said and what…(Carer of P14)*
	*Participant: Oh god they’re all doing my head in because they can’t get time off to come with me so, ‘what about this and…’ (P14)*
Carer interpretation of HF	*The breathing, the coughing, they said it was on his lungs. His lungs weren’t… because his heart wasn’t beating properly all the gunge was going on to his lungs. (Carer of P16)*
Carers clarify explanations of HF to participants	*Because the [carer - nurse] came with me ‘cause she understands the old… medical jargon what they like to… talk on, you know sometimes. And the ordinary patient can’t understand what they’re talking about. She can and she’ll refer it back to us after. (P16).*

### Tensions between noticing and normalising (before seeking medical help)

While participants and their carers acknowledged that carers were often able to perceive symptoms even when the participant was not, particularly in the case of breathlessness which participants either did not notice or ignored, there was not a straightforward relationship between this and help-seeking. Carers often instigated or supported participants’ efforts to normalise symptoms when they first emerged, and a frequent assumption was that symptoms were a sign of ageing rather than of disease. Some carers compared the participant’s symptoms to their own ageing, suggesting that the two were similar and therefore the participant’s symptoms were not anything to be concerned about.

Normalisation broke down when symptoms did not follow the trajectory that the carer expected. In some cases, there was a sudden onset of severe symptoms and in others, carers became concerned when symptoms were particularly prolonged.

### From noticing to sanctioning (seeking medical help)

In many instances, it was carers’ insistence in their concern about the symptoms they had noticed that led participants to finally seek medical help and enable the start of the medical diagnosis process.

Although our data do not provide frequencies, stories of ‘noticing’ and ‘sanctioning’ often involved other family members, particularly grown up children, despite interviews indicating that spouses also noticed participants’ symptoms. They were reported to notice significant deterioration in health and function which given the insidious nature of the onset of HF, with a gradual decline in function, was perhaps less likely to be noticed by a cohabiting spouse or partner.

Even when help-seeking was sanctioned by family members, it could still be seriously delayed when participants reported that they were reluctant to follow the advice of their carers to seek medical help or go to hospital. In some cases, the participant became acutely unwell before the carers’ concern influenced the help-seeking behaviour of the participant.

### Coordinating care and self-care (getting the diagnosis)

During the diagnostic process, many informal carers felt that they took on a greater or lesser role of ‘care coordinator’ for the participant’s healthcare. This included keeping records of GP and hospital visits, filing important letters and arranging appointments. The care coordinator role was experienced as burdensome for some carers as it required a lot of time and effort.

The care coordinator role also entailed advocating for participants. Some carers felt that participants’ symptoms were not being adequately addressed and they advocated for them to ensure they received the healthcare they thought they needed. For example, one carer phoned the secretary of her partner’s consultant every day for several weeks to request an earlier appointment because she felt he was so unwell.

Carers also facilitated independence and encouraged self-care by offering both practical and emotional support to participants throughout the diagnostic process. This included providing equipment for participants whose symptoms made day-to-day life more difficult, for example the provision of a bannister for one participant who had difficulty getting upstairs. Carers also advised participants on self-care; for example, suggesting they rest in circumstances when their symptoms worsened.

### Making sense of information (understanding the diagnosis)

Through accompanying participants to medical consultations and being present with them in many other areas of their lives, carers gained valuable information about the participant’s illness within its social context and tried to understand the potential impact of the HF diagnosis.

Carers’ understanding of the diagnosis and treatment was generally at least as good as, and sometimes better than, the participant’s understanding. If they were unable to accompany the participant to a consultation, due to work commitments for example, carers expressed a desire to know more about the information given in appointments. Some carers had developed useful explanations for why symptoms were occurring and how they linked to HF from information provided by healthcare professionals. Carers with a medical background, for example nurses and physiotherapists, played a particular role by clarifying aspects of the diagnosis that participants and other carers did not understand.

In many cases, the diagnosis led to anxiety for both the carer and participant reflecting a common lack of understanding about what HF was. For instance, there was particular alarm around ejection fraction: two participants had been told that their ejection fraction was 20%, and carers and participants assumed this meant that only 20% of the heart was working and were unaware that a normal ejection fraction is 50% or above.

## Discussion

### Summary

In this analysis, it is clear that both people with a diagnosis of HF and their carers perceive the carer role to be important in the process of diagnosis of HF for all participants. Carers noticed and responded to symptoms, even when the participant themselves did not realise anything was wrong. The proximity of the relationship of the carer to the participant had an impact on the nature of this response, in terms of whether noticing symptoms resulted in normalization of symptoms or sanctioning of help seeking: for instance, adult children who were less proximal were often reported to push for the participant to seek medical help promptly when they noticed significant deterioration, while spouses were more likely to initially normalise symptoms as normal signs of ageing. Once help was sought, carers supported the participant through the diagnostic journey emotionally and practically and were at least as knowledgeable as the participant regarding the diagnosis, although understanding of some aspects of HF was limited. Several carers were actively involved in organising and co-ordinating the participants’ professional care.

### Strengths and limitations

Several studies have examined the experience of informal carers of patients with established HF, but this study is unique in focusing on the role of carers before and during the HF diagnostic process. The data offer an insight into the influence informal carers had at key stages of the diagnostic journey. The original purpose of the interviews was to gain an understanding of patient experiences during this period, and this secondary analysis describes the ways that the role of carers was understood by the families involved.

Secondary analysis of any existing dataset has limitations but can still be useful to inform further research and policy [18]. The aim of the original study was to explore the patient experience and recruited only people with a new diagnosis of HF. Carers were present during participant interviews in some cases but separate interviews with carers alone were not part of the design. The study did not explore whether relatives would have identified themselves as informal carers or if the participants would have identified them as their carers. However, the role that relatives played in the participants’ lives resembled the accepted definition of an informal carer: anyone who cares, unpaid, for a friend or family member who due to illness cannot cope without support [19]. A further limitation is that carers were not present at all interviews and we do not know whether data saturation, for the purposes of this study, occurred. The topic guide was appropriately structured to allow the participant to describe freely factors that were important in the diagnostic process and the importance of relatives as informal carers was evident in almost all interviews. The contribution of carers present during interviews was encouraged. This secondary analysis has allowed exploration of the role of carers from an existing dataset but did not allow the deep and thorough approach advocated by Silverman to the conduct and analysis of an interview study specifically recruiting carers [20]. A further study, with the aim of capturing the views and experiences of carers would ensure data saturation and allow more sophisticated analysis.

### Comparison with existing literature

Previous research studied informal carer’s contributions to self-care for people with established HF [6]. Informal carers responded to certain symptoms by calling the clinician and facilitating clinic appointments, and were also involved in navigating the healthcare system and providing understanding and support.

The theme of normalisation of symptoms secondary to ageing has also been described in interviews with informal carers of patients with Alzheimer’s disease [21]. Public perception that memory loss is a normal consequence of ageing, rather than symptomatic of dementia, is commonplace [22]. Krull et al. reported that informal carers’ normalisations broke down due to a pivotal event in which symptoms could no longer be normalised. Intervention from ‘outsiders’ – individuals trusted by the informal carer but not involved in their day-to-day life, who observed and reported the symptoms of the carer’s loved one – was also influential in breaking down normalisations.

Normalisation of symptoms, as well as being due to a lack of understanding of HF symptoms and belief that they are part of normal ageing, may be a mechanism of collusion between participants and their spouses. This has also been described in the case of dementia symptoms [23]. Wingham et al recently reported that carers experience anguish in the transition to becoming an informal carer of someone with HF due to a changing sense of self [24]. Normalisation may unintentionally be used to delay diagnosis because carers and people with HF symptoms do not want their daily roles to change. Adult children who do not cohabit with their parents may be less likely to expect a significant role change to come with diagnosis so are perhaps less likely to be involved in collusion.

An important finding of our study was that the carer’s recognition of and concern about symptoms led participants to seek medical help. Several studies have implicated witnesses of symptoms in help-seeking, a process that is often termed sanctioning [25]. Patel et al interviewed 88 patients diagnosed with deteriorating chronic HF from a Swedish community and described a ‘wait and see’ strategy used by HF patients to delay hospital admission. In 15% of cases, patients were sent to hospital by their spouses, children or home carers, who noticed something was seriously wrong despite strong denials from patients [26]. It was clear from our interviews that some participants did not want to go to hospital for their symptoms and disregarded the advice of carers, and sometimes carers would take control and take them to see a medical practitioner.

Furthermore, despite Patel et al studying patients who had already been diagnosed with HF, participants in the study still tended to normalise exacerbations of HF symptoms as being part of old age [26]. This suggests that better communication to ensure patient understanding of HF diagnosis is required. Other studies also found that patients with organ failure and their carers have an inadequate understanding of their diagnoses [27]. In a comparative study of the experiences of patients with cancer, organ failure and frailty and their carers, Kendall et al [28] reported that patients with cancer, and their carers, had a much more detailed knowledge of their illness in comparison to patients with organ failure.

The gender of carers may also have influenced our results. Nine out of the ten carers present during the interviews were female. The role of women in caregiving has been previously explored in carers of people with dementia. Tolhurst et al. describe how gendered meanings of care can affect the experience of carers [29]. They describe the challenges of ‘caring’ being defined as a natural female role, associated with nurturing and maternal values, and the impact this can have on female self-identity. The role of gender may have had some impact on the findings of our study and prospective research, recruiting both male and female carers, is needed to explore this further.

### Implications for research and practice

This study found differences between the roles of spouses and children as carers of people with HF which could have implications for practice. The spouse of the person with symptoms tended to be less active in seeking medical help compared to adult children, even when they notice the decline that comes with HF. Public awareness of the symptoms of HF and understanding of its causes has been repeatedly found to be inadequate, with a large proportion of the European public unable to recognise breathlessness and swollen ankles as symptoms of HF, and often thinking that HF is a normal consequence of ageing [30].

A qualitative synthesis of help-seeking in the event of cancer symptoms found similar triggers to this study, including ‘discussion of symptoms with friends and family’ [31]. However, the study also found that ‘specific well-known symptoms’ and ‘knowledge of cancer symptoms and awareness of risk’ were triggers for help-seeking. Any knowledge of HF symptoms before diagnosis was absent from the interviews in our study. Public health initiatives such as the Be Clear on Cancer campaign have focused on detection of cancer symptoms [32]. and similar initiatives for HF may have an impact on increasing awareness of HF symptoms.

## Conclusion

Increased public awareness of HF symptoms for adults of all ages could lead to timelier HF diagnosis. Partners and adult children should be empowered to encourage people with HF symptoms to seek medical help. The crucial role of carers in understanding a HF diagnosis and coordinating patients’ healthcare should be incorporated into future diagnostic pathways. The needs and challenges faced by carers during the HF diagnostic pathway should be explored in further research.

## Abbreviation

HF: Heart failure
